# Characteristics of people living with undiagnosed dementia: findings from the CFAS Wales study

**DOI:** 10.1186/s12877-022-03086-4

**Published:** 2022-05-10

**Authors:** Laura D. Gamble, Fiona E. Matthews, Ian R. Jones, Alex E. Hillman, Bob Woods, Catherine A. Macleod, Anthony Martyr, Rachel Collins, Claire Pentecost, Jennifer M. Rusted, Linda Clare

**Affiliations:** 1 Population Health Sciences Institute, Newcastle University, Newcastle upon Tyne, UK; 2 Wales Institute for Social and Economic Research and Data, Cardiff University, Cardiff, UK; 3 Department of Public Health, Policy and Social Sciences, College of Health and Human Sciences, Swansea University, Swansea, UK; 4 Dementia Services Development Centre Wales, School of Medical and Health Sciences, Bangor University, Bangor, UK; 5 REACH: The Centre for Research in Ageing and Cognitive Health, University of Exeter Medical School, Exeter, UK; 6 School of Psychology, University of Sussex, Brighton, UK; 7 NIHR Applied Research Collaboration South-West Peninsula, Exeter, UK

**Keywords:** Alzheimer’s, Depression, Sleep, Apathy, Hallucinations, Neuropsychiatric symptoms, Diagnosis

## Abstract

**Background:**

Many people living with dementia remain undiagnosed, with diagnosis usually occurring long after signs and symptoms are present. A timely diagnosis is important for the wellbeing of the person living with dementia and the family, allowing them to plan and have access to support services sooner. The aim of this study was to identify demographic characteristics and neuropsychiatric symptoms associated with being undiagnosed, which may help clinicians be more aware of signs that could be indicative of early-stage or undetected dementia.

**Methods:**

This cross-sectional study uses data from waves 1 and 2 (two years apart) of the Cognitive Function and Ageing Studies Wales (CFAS Wales). CFAS Wales participants were included who had a study assessment of dementia, as determined by the Automated Geriatric Examination for Computer Assisted Taxonomy (AGECAT) algorithm and by expert assessment, and who had had their primary care records checked for a clinical diagnosis of dementia. We identified 19 people with a diagnosis of dementia and 105 people living with undiagnosed dementia, and explored demographic characteristics and the presence or absence of a range of neuropsychiatric symptoms in the undiagnosed population using logistic regression.

**Results:**

Findings suggest that people living with dementia who have better cognition, have more years of education, or live in more deprived areas are less likely to have a diagnosis. In terms of neuropsychiatric symptoms, depression and sleep problems were associated with being undiagnosed. Apathy was common across all people living with dementia, but those with a diagnosis were more likely to have severe apathy.

**Conclusions:**

This study has clinical practice implications as the findings may help clinicians be more aware of characteristics and symptoms of people who are undiagnosed or who are at greater risk of remaining undiagnosed, enabling them to be more vigilant in picking up signs of dementia at an earlier stage.

**Supplementary Information:**

The online version contains supplementary material available at 10.1186/s12877-022-03086-4.

## Background

The social and economic impact of dementia is one of the major challenges of this century [1]. It is a key priority in health and social care planning across the world, with an increasing emphasis on timely diagnosis and appropriate support throughout the trajectory of the condition [2]. According to the World Health Organization (2020), more than 55 million people worldwide have a diagnosis of dementia [3], and the number of people living with dementia is projected to double by 2040 [4]. However, even in high income countries with advanced universal healthcare systems diagnosis rates remain relatively low, with estimates of only 20–50% of cases documented in primary care [5]. In 2018, of the 850,000 people estimated to be living with dementia in the United Kingdom (UK) only 537,000 were diagnosed by primary care physicians or through memory services [6].

Dementia is characterised by a decline in one or more cognitive domains including learning and memory, language, executive function, complex attention, perceptual-motor function and social recognition [7]. These declines lead to increasing functional impairment and significant disabilities that impact on daily life [8–10]. Dementia is often diagnosed long after the symptoms and signs are evident. One study found that people who developed dementia performed worse in complex activities of daily living 10 years before their diagnosis [11, 12]. Although symptoms will typically get worse as the condition progresses, timely diagnosis allows the person living with dementia to maintain control of decision making and planning, and helps both the person living with dementia and the family to access appropriate care and support sooner [13–15]. It may lead to a delay in dependency and subsequently delay entry into residential care [5, 16, 17].

Reasons for people remaining undiagnosed have been explored in some qualitative studies and are complex [18, 19]. Fear of stigmatization, which can impact on psychosocial wellbeing and can cause people to feel marginalised, is a major factor that contributes to a delay in seeking a diagnosis [17, 20, 21]. People may also be reluctant to seek a diagnosis because of fear about the future, the belief that any increased support they might need is unavailable, individual perceptions that there are no treatments available and because they or their families believe they can cope and retain independence [17, 18]. Lack of awareness of dementia amongst the general public is another barrier to getting a diagnosis [3], and sometimes people are unaware themselves that they have a problem or may be in denial [22].

Identification of the signs and symptoms of dementia can also be a challenge in a clinical setting [23]. The criteria for a dementia diagnosis vary but typically require an impairment in at least 2 aspects of cognitive function and an associated impact on activities of daily living [7, 24]. Diagnosis is a long process that can sometimes take many years [3]. Detection of dementia by primary care physicians is low [25], particularly in the early stages of the condition where the symptoms can be difficult to distinguish from normal cognitive ageing and from short-term loss of function secondary to other problems such as depression [3], and also because of the slow progression in the case of the most prevalent type of dementia, Alzheimer’s disease [26]. Due to the potential risks of over-diagnosis, clinicians may also be cautious in order to avoid a misdiagnosis of dementia [27]. Dementia is heterogeneous with over 100 sub-types, each of which can have different profiles of cognitive and non-cognitive symptoms, which adds to the difficulties in diagnosing the condition [17]. Finally, whilst the first point of contact is usually a primary care physician, lack of awareness or confidence among practitioners and limited training regarding dementia can be significant barriers to receiving a diagnosis [3].

Although numerous factors have been proposed as possibly relevant to whether or not people living with dementia receive a diagnosis, to date little is known about the characteristics and features of those living with undiagnosed dementia, with just a handful of studies providing quantitative evidence for the person-level characteristics that may facilitate or prevent people living with dementia from seeking help or receiving a diagnosis [28, 29]. Previous studies have looked for associations between demographics and diagnosis but cohorts differ by geographic location, cohort compositions or demographics, and the way in which dementia is assessed, and many findings are inconsistent.

During the course of their dementia, most people will experience some neuropsychiatric symptoms such as hallucinations, delusions, sleep problems, apathy, irritability, depression, anxiety, and elation. These symptoms are associated with worsening condition and burden, and symptom severity correlates with institutionalisation [30]. Whilst neuropsychiatric symptoms have been shown to be more prevalent in people living with dementia compared to those without [31], few studies have looked at neuropsychiatric symptoms and their association with diagnosis. Two studies found no association with diagnosis and depression or anxiety [17, 32], and a US based study found that apathy, aberrant motor behaviour, and agitation were more common in those with a diagnosis [33].

This exploratory study examines a cohort of people living in Wales, a constituent nation of the UK with the lowest reported rates of dementia diagnosis (53%) [6]. True population-based studies looking at undiagnosed dementia cases are rare. This study is unique in that it determines whether participants have a formal dementia diagnosis using an official dementia register, it uses a standardised validated assessment of dementia in all study participants, and it investigates additional measures that other studies have not taken into account. The aims are first to identify demographic characteristics associated with being undiagnosed and second to determine whether there are neuropsychiatric symptoms present in people living with undiagnosed dementia, and if so, which are the most prevalent.

## Methods

### Study design

This study uses data from a population-based longitudinal cohort study of people living in Wales: Cognitive Function and Ageing Study Wales (CFAS Wales). CFAS Wales examines risk factors and health outcomes in people living in Wales aged 65 or over. The study was conducted across two locations; one rural (Gwynedd and Ynys Môn) and one urban (Neath Port Talbot). People were randomly selected from primary care practice lists and stratified into two equally sized age groups (65–74 and ≥ 75). Following initial checks with family practitioners to determine whether there was any reason why a participant should not be contacted (for example if receiving end of life care), selected participants were sent an invitation and information sheet about the study in the mail. Invitations were followed up with a home visit by trained interviewers in the following weeks to discuss the study in more detail. On the day of the interview interviewers read through the information sheet and consent forms with participants, discussing the information and answering any questions to ensure participants understood the information and were making an informed decision. Participants were asked to sign separate consent forms for each part of the study to facilitate personal choice about which parts of the study to participate in. There were two waves of data collection. Baseline data were collected between 2011 and 2014 and follow-up was two years later (2013 to 2016). Ethical approval for data collection was granted by the NHS North Wales – West Research Ethics Committee (REC Ref No: 10/Wno01/37; IRAS Project No: 40092).

The participant interview covered the following categories: demographic characteristics, lifestyle variables, health status, functional limitations, cognitive function, social support and social networks, and measures of hearing and visual impairment. Study diagnosis of dementia, anxiety and depression was determined using the AGECAT diagnostic algorithm which draws on respondent and observer ratings [34, 35]. The AGECAT algorithm is a standardised validated assessment of eight syndromes, including dementia, depression and anxiety, with all participants allotted to one of six levels of confidence of the diagnosis of each syndrome. The algorithm is described extensively by Copeland et al. [35]. Where a participant lacked capacity, an informant, usually a spouse or relative, was consulted as to whether the person would want to take part. In these cases, an informant interview was conducted for the refinement of the study diagnosis, to provide proxy information and to provide informant ratings. Where there was insufficient information for the AGECAT algorithm, a medically-qualified member of the study team reviewed data from informant interviews and interviewer vignettes to consider whether a diagnosis of dementia was justified. The data are stored on secure computer systems at the Secure Data Holding Service, Clinical School, University of Cambridge, along with data from all the CFAS studies, with the University of Cambridge as the Data Controller, and are regulated under the University of Cambridge clinical governance procedures. CFAS Wales data are deposited with the UK Data Service [36]. Registered users are only provided with data from which identifiable information of study participants has been removed.

### Definition of the study sample

From the 3593 people interviewed for CFAS Wales at baseline, of interest were those people who had dementia determined through an assessment that was a part of the study (‘study dementia’), and had also had their primary care records checked for a clinical diagnosis of dementia. The study was cross-sectional, but data from either wave 1 or wave 2 were used dependent on the person’s dementia status at a given wave. ‘Study dementia’ was determined using the AGECAT diagnostic algorithm for 3484 people and by expert assessment for 109 people at wave 1, and assessed again for those followed up at wave 2. An official clinical diagnosis of dementia (‘diagnosed dementia’) was determined by checking primary care records, which included the dementia register. The dementia register is a list of patients with a dementia diagnosis which practices were required to set up and maintain as part of their contract with the UK Government. Of the people interviewed, 2771 consented to having their medical notes reviewed and their primary care records were checked for a diagnosis of dementia. Two hundred and six were excluded due to undetermined diagnostic information as they had either died or were no longer registered or active at the primary care practice. Of the remaining 2565 participants, 19 were identified as having ‘diagnosed dementia’, having both study dementia and a clinical diagnosis of dementia recorded at the date of the study interview (either at wave 1 or wave 2). For people living with diagnosed dementia, the wave used in the analysis was the wave after the date of diagnosis. In some cases, a participant did not have a study diagnosis at wave 1 but did at wave 2 (incident cases), and in these cases the responses from wave 2 were used in the analysis. Twelve of the 19 people living with diagnosed dementia had an informant taking part. Ninety people without a clinical diagnosis of dementia met study criteria for dementia. An additional 11 potential undiagnosed participants were identified who were not on the dementia register but whose cognitive function appeared to improve over time, meeting study criteria for dementia at wave 1 but not at wave 2. Mini-Mental State Examination (MMSE) [37] and AGECAT outputs were checked and compared for each wave and three of these 11 people were included where data were indicative of dementia at wave 2; the remaining eight participants were excluded. Twelve people had study dementia and were clinically diagnosed with dementia but at a date later than the study interview so were ‘undiagnosed’ during the study assessment. Therefore, 105 people living with undiagnosed dementia were included in the study. For the undiagnosed dementia group, the wave used in the analysis was the one where study dementia was observed and where the interview date was before any date of diagnosis. If there was no clinical diagnosis of dementia at either wave, then the latest wave where the participant had met criteria for study dementia was used. Fifty nine of the 105 people living with undiagnosed dementia had an informant taking part. Of the remaining people, 2392 neither had study dementia nor were on the dementia register and 41 did not have study dementia but were on the dementia register; these people were excluded from this study. See flowchart in Supplementary Fig. 1.

By linking primary care records with epidemiological data, we explored aspects of the population without a clinical diagnosis, including demographic characteristics and neuropsychiatric symptoms.

### Measures

Comparisons were made using measures that covered demographic information and neuropsychiatric symptoms. Further details on the measures used can be found in Supplementary Table 1. The Welsh Index of Multiple Deprivation 2014 (WIMD) was used to measure social deprivation, and was categorised into three quantiles based on the rank of the Lower Layer Super Output Area (LSOA) where the person lived [38]. Years of education was collected and used in the analysis. MMSE was used as a measure of cognitive ability, and was originally split into three categories (< 18, 18–21, and 22–30) as previously described [31]. However, due to low numbers in the 22–30 category, the 18–21 and 22–30 categories were combined. For those with missing MMSE scores, the most likely cause is very severe cognitive impairment, so these were assigned to the lowest category (< 18) [31]. The number of health conditions was computed as previously described and in line with the scoring system of the Charlson Comorbidity Index [39, 40]. Neuropsychiatric symptoms were assessed in this study by asking about the presence of hallucinations, sleep problems, apathy, delusions, irritability, depression, anxiety, and elation (see Supplementary Table 1). Neuropsychiatric symptom questions that concerned the person living with dementia were self-rated by the person living with dementia and informant-rated by the carer where available, and included observations made by the researcher. For hallucinations, apathy, delusions, and irritability a second analysis was conducted when more severe symptoms were reported. Depression was determined using the AGECAT algorithm or a clinical diagnosis of depression, or both, and anxiety was measured using just the AGECAT algorithm [34, 41].

### Statistical analysis

Analyses were conducted using R Statistical Software. Descriptive information was reported for people living with diagnosed or undiagnosed dementia and possible predictors of undiagnosed dementia were identified a priori and tested individually using logistic regression. Multiple imputation was conducted on variables at the item-level to allow, where appropriate, for missing response data using the *mice* package [42]. Total scores and prevalence of neuropsychiatric symptoms were computed following imputation, and estimates from 25 imputed datasets were combined according to Rubin’s rules [43]. Variables not considered to have data missing at random were not imputed, such as informant ratings. Univariate analyses were conducted, followed by analyses adjusted for age, sex, and binary MMSE score (< 18 vs ≥ 18), and further adjustment for depression. Odds ratios (OR) are reported alongside their confidence intervals (CI). Further confounders were not considered due to the small sample size and due to lack of evidence for association.

## Results

The sample comprised 124 people; 105 were undiagnosed and 19 were diagnosed with dementia. Table 1 shows the demographic characteristics and prevalence of each neuropsychiatric symptom in the overall sample according to diagnostic status. Briefly, the median age of the cohort was 82 years, and approximately half the cohort were female. Almost half were married, 31% lived alone and 14% lived in residential care. Almost half of the cohort had an MMSE score below 18 and the average number of health conditions in addition to dementia was approximately three. The most prevalent neuropsychiatric symptoms were apathy (85%) and irritability (38%), whereas the least prevalent were hallucinations (12%), anxiety (8%), and elation (3%). For those who reported neuropsychiatric symptoms that were judged to be severe, 38% experienced symptoms of apathy, whereas severe symptoms of hallucinations, irritability, and delusions were experienced by fewer than 10% of people.

**Table 1:** Demographic characteristics and neuropsychiatric symptoms of people in the CFAS Wales study sub-sample

		**Total Sample (*n* = 124)**	**Diagnosed**	**Undiagnosed**
			**Dementia (*n* = 19)**	**Dementia (*n* = 105)**
Sex (n, %)	Male	61 (49.2%)	11 (57.9%)	50 (47.6%)
	Female	63 (50.8%)	8 (42.1%)	55 (52.4%)
Age (mean, SD)		81.5 (7.2)	83.1 (6.5)	81.2 (7.3)
Education years (mean, SD)		10.4 (1.8)	9.6 (6.5)	10.5 (7.3)
Missing (N)		3	1	2
Marital status (N, %)	Married/Cohabiting	59 (48.3%)	10 (55.6%)	49 (47.1%)
	Widowed/Divorced/Single	63 (51.6%)	8 (44.4%)	55 (52.9%)
	Missing	2	1	1
Living situation (N, %)	Lives alone	38 (31.1%)	5 (26.3%)	33 (32.0%)
	Lives with someone	67 (54.9%)	10 (52.6%)	57 (55.3%)
	In residential care	17 (13.9%)	4 (21.1%)	13 (12.6%)
	Missing	2	0	2
MMSE (N, %)	MMSE < 18 and missing	58 (46.8%)	15 (78.9%)	43 (41.0%)
	MMSE 18–30	66 (53.2%)	4 (21.1%)	62 (59.0%)
Deprivation (N, %)	1 – Least deprived	41 (33.1%)	9 (47.4%)	32 (30.5%)
	2	41 (33.1%)	7 (36.8%)	34 (32.4%)
	3 – Most deprived	42 (33.9%)	3 (15.8%)	39 (37.1%)
Health conditions (mean, SD)		3.2 (2.2)	2.8 (1.7)	3.2 (2.2)
Missing (N)		30	7	23
*Neuropsychiatric symptoms (present)*				
Hallucination (N, %)	No	106 (87.6%)	16 (84.2%)	90 (88.2%)
	Yes	15 (12.4%)	3 (15.8%)	12 (11.8%)
	Missing	3	0	3
Sleep problems (N, %)	No	70 (60.9%)	14 (77.7%)	56 (57.7%)
	Yes	45 (39.1%)	4 (22.2%)	41 (42.3%)
	Missing	9	1	8
Apathy (N, %)	No	18 (15.5%)	2 (11.1%)	16 (16.3%)
	Yes	98 (84.5%)	16 (88.9%)	82 (83.7%)
	Missing	8	1	7
Delusions (N, %)	No	90 (77.6%)	12 (66.7%)	78 (79.6%)
	Yes	26 (22.4%)	6 (33.3%)	20 (20.4%)
	Missing	8	1	7
Irritability (N, %)	No	76 (61.8%)	11 (57.9%)	65 (62.5%)
	Yes	47 (38.2%)	8 (42.1%)	39 (37.5%)
	Missing	1	0	1
Depression (N, %)	No	84 (73.0%)	16 (94.1%)	68 (68.7%)
	Yes	31 (27.0%)	1 (5.9%)	30 (30.6%)
	Missing	9	2	7
Anxiety (N, %)	No	105 (92.1%)	17 (100.0%)	88 (90.7%)
	Yes	9 (7.9%)	0 (0.0%)	9 (9.3%)
	Missing	10	2	8
Elation (N, %)	No	105 (97.2%)	16 (94.1%)	89 (97.8%)
	Yes	3 (2.8%)	1 (5.9%)	2 (2.2%)
	Missing	16	2	14
*Neuropsychiatric symptoms (severe)*				
Hallucination (N, %)	No	111 (92.5%)	16 (84.2%)	95 (93.1%)
	Yes	10 (8.3%)	3 (15.8%)	7 (6.9%)
	Missing	3	0	3
Apathy (N, %)	No	72 (62.1%)	8 (44.4%)	64 (65.3%)
	Yes	44 (37.9%)	10 (55.6%)	34 (34.7%)
	Missing	8	1	7
Delusions (N, %)	No	107 (92.2%)	15 (83.3%)	92 (93.9%)
	Yes	9 (7.8%)	3 (16.7%)	6 (6.1%)
	Missing	8	1	7
Irritability (N, %)	No	112 (91.1%)	17 (89.5%)	95 (91.3%)
	Yes	11 (8.9%)	2 (10.5%)	9 (8.7%)
	Missing	1	0	1

*Note*: *MMSE* Mini-Mental State Examination, *SD* standard deviation

Logistic regressions explored the relationship between undiagnosed dementia, neuropsychiatric symptoms, and demographic characteristics (Table 2). Three models were conducted for each measure; an unadjusted model (Model 1), a model adjusted for age, sex and MMSE score (Model 2), and a model further adjusted for depression (Model 3).

**Table 2:** Regression to identify associations between demographics and neuropsychiatric symptoms, and undiagnosed dementia

		Model 1				Model 2			Model 3		
		Unadjusted				Adjusted			Adjusted		
		OR (95% CI)			*p*	OR (95% CI)		*p*	OR (95% CI)	*p*	
Sex (female)		1.51 (0.56 – 4.10)			0.413	-					
Age (years)		0.96 (0.90 – 1.03)			0.294	-					
Education (years)		1.51 (0.93 – 2.54)			0.097	1.67 (0.93 – 2.98)		0.088			
Marital status (married/cohabiting)		0.80 (0.30 – 2.16)			0.647	0.65 (0.22 – 1.94)		0.433			
Living Situation	Lives with others	Ref			-	Ref		-			
	Lives alone	1.15 (0.36 – 3.69)			0.831	0.99 (0.29 – 3.44)		0.978			
	In residential care	0.58 (0.15 – 2.16)			0.402	0.96 (0.23 – 4.00)		0.931			
MMSE	< 18 and missing	0.18 (0.06 – 0.60)			**0.005**	-					
Deprivation	1 – Least deprived	Ref			-	Ref		-			
	2	1.37 (0.45 – 4.15)			0.579	1.58 (0.48 – 5.26)		0.452			
	3 – Most deprived	3.66 (0.90 – 14.86)			0.070	4.34 (0.98 – 19.24)		0.054			
Number of health conditions		1.12 (0.82 – 1.54)			0.473	1.11 (0.77 – 1.62)		0.574			
* Neuropsychiatric symptoms (present)*											
Hallucinations		0.60 (0.22 – 1.66)		0.311		0.85 (0.29 – 2.56)		0.739	0.65 (0.21 – 2.07)		0.440
Sleep problems		1.71 (0.56 – 5.26)		0.330		2.46 (0.70 – 8.58)		0.145	1.93 (0.51 – 7.33)		0.321
Apathy		0.36 (0.04 – 2.95)		0.340		0.37 (0.04 – 3.32)		0.386	0.28 (0.03 – 2.57)		0.269
Delusions		0.44 (0.16 – 1.19)		0.092		0.64 (0.22 – 1.88)		0.374	0.44 (0.14 – 1.41)		0.147
Irritability		0.66 (0.24 – 1.84)		0.424		0.83 (0.28 – 2.46)		0.732	0.68 (0.22 – 2.08)		0.497
Depression		3.07 (0.81 – 11.63)		0.097		3.05 (0.76 – 12.16)		0.111			
Anxiety		1.65 (0.31 – 8.83)		0.517		2.06 (0.36 – 11.72)		0.399	0.80 (0.08 – 7.72)		0.938
Elation		0.94 (0.24 – 3.66)		0.941		1.32 (0.31 – 5.60)		0.687	0.67 (0.13 – 3.48)		0.652
* Neuropsychiatric symptoms (severe/persistent symptoms only)*											
Hallucinations		0.47 (0.17 – 1.31)	0.143			0.72 (0.24 – 2.17)	0.544		0.52 (0.16 – 1.68)		0.269
Apathy		0.39 (0.13 – 1.15)	0.102			0.49 (0.16 – 1.50)	0.236		0.32 (0.10 – 1.05)		0.071
Delusions		0.38 (0.13 – 1.07)	0.064			0.62 (0.20 – 1.91)	0.392		0.47 (0.14 – 1.54)		0.212
Irritability		0.58 (0.20 – 1.70)	0.308			0.81 (0.26 – 2.52)	0.709		0.59 (0.18 – 1.95)		0.384

*Note*: *MMSE* Mini-Mental State Examination, *OR* Odds ratio, *CI* Confidence intervals. Model 1 is unadjusted. Model 2 is adjusted for sex, age and MMSE. Model 3 is adjusted for sex, age, MMSE, and depression. Bold indicates that *P* < 0.05

### Demographic characteristics and cognition

Compared to those without a clinical dementia diagnosis, in the diagnosed dementia group there were more males (58% vs 48%), a higher mean age (83 vs 81), more married people (56% vs 47%), fewer people who lived alone (26% vs 32%) and more people who lived in residential care (21% vs 13%). The wide confidence intervals of the odds ratios indicate uncertainty in these findings. Despite wide confidence intervals there was some indication that those with more years of education were less likely to be diagnosed (OR 1.67, 95% CI: 0.93, 2.98), as were those who were from more deprived areas (most deprived vs. least deprived; OR 4.34, 95% CI: 0.98, 19.24). People with an MMSE score below 18 were more likely to have a clinical diagnosis of dementia (OR 0.18, 95% CI: 0.06, 0.60), indicating that people with poorer cognitive ability were more likely to be diagnosed. The number of health conditions was not associated with having received a diagnosis of dementia.

### Neuropsychiatric symptoms

Findings indicate that those with depression (OR 3.05, 95% CI: 0.76, 12.16) and those with sleeping problems (OR 2.46, 95% CI: 0.70, 8.58) may be more likely to be undiagnosed. Apathy (mild or severe) was present in the majority of people suggesting that this is common in people living with dementia; however, there was some indication that those with severe symptoms may be more likely to be diagnosed with dementia, and this was strengthened after adjusting for depression (OR 0.32, 95% CI 0.10, 1.05). Odds ratios suggest that hallucinations, delusions, and irritability may be less likely in people living with undiagnosed dementia but due to small sample sizes and wide confidence intervals there is greater uncertainty in these findings. Evidence is lacking for a link between anxiety and diagnosis, particularly after adjusting for depression, and there was little evidence of a link between symptoms of elation and a diagnosis of dementia, although few participants in this study experienced elation.

## Discussion

Understanding the factors influencing the levels of undiagnosed dementia is important for improving detection rates, a key target for the Welsh and other UK governments [44, 45]. Improved detection rates are important for the wellbeing of people living with dementia and their families through improved choice and access to appropriate support services as well as for health policy and planning. Identification of the characteristics of people who have undiagnosed dementia is challenging and this is one of very few studies to systematically explore differences between individuals with diagnosed and undiagnosed dementia. We have used data from a large cohort, CFAS Wales, to address this issue, focusing on people living in Wales where dementia diagnosis rates are the poorest in the UK [4].

Our study found, in agreement with other studies, that those with better cognition are less likely to have a diagnosis [17, 32, 33, 46, 47]. This is to be expected as dementia is typically diagnosed when cognitive impairments have become severe enough to impact on social or occupational functioning [48]. We found no association between age and sex and the likelihood of having a diagnosis, nor was there an association between diagnosis and either living alone or marital status. Several other studies have found that those who are older than 90 are less likely to be diagnosed [33, 46, 47] whereas a meta-analysis found those who are younger are less likely to be diagnosed [49]. Aldus et al.suggest that both those over 90, and those under 70 are less likely to be diagnosed [17]. The mean age was higher in our diagnosed sample compared to our undiagnosed sample, but we did not categorise age into groups due to small sample size and it is likely that we found no relationship in our study due to lack of statistical power. Findings in other studies relating to sex and diagnosis are inconsistent. Some studies found that women were more likely to have a diagnosis [32, 33, 49]. However, other studies, including a UK based study by Aldus et al., similarly found no association with sex [17, 50]. Whilst an association between living alone and being undiagnosed has been found in other studies [33, 46, 47], and we can speculate that these people may be less inclined to seek help, we did not find an association with living alone. However, approximately 70% of our sample did not live alone and again our study may have lacked statistical power to support a finding. Diagnosis rates can be influenced by social or geographical factors, including socioeconomic status and rural or urban settings [45]. Our findings indicate that more years of schooling increased the risk of being undiagnosed. This finding is supported by numerous studies that show lower education is a risk for dementia later in life [51]. People with more education may be higher performers, making earlier signs of dementia more difficult to detect on standardised tests since they do not reach the threshold required for a dementia diagnosis [52]. Despite our findings being well supported by theory, Aldus et al.found no association between education years and diagnosis in the UK [17], and two other studies found that those with the lowest levels of education (less than 9 years, or less than high school) were less likely to have a diagnosis [32, 33]. Historical changes in education systems may impact on results. For example, in England and Wales those who left school prior to 1947, when the school leaving age was raised to 15 following the 1944 Education Act, would have had fewer years of compulsory education than those who left school after 1947. As a result, assuming they were educated in England or Wales, older people in the study may have had fewer years of education. Because the mean age is slightly higher in the group living with diagnosed dementia, this is likely to contribute to the observation of fewer years of education in this group. However, the finding that those living with undiagnosed dementia are likely to have more years of education remains following adjustment for age. Perhaps counterintuitively to our finding for education and suggestive of a different pathway, there was also evidence in our study that under-diagnosis is linked to area-level deprivation. Our findings here may be related to inequalities in provision of services, or may be due to differences in help-seeking behaviours of people in deprived areas who may be less aware of the potential benefits of seeking help [53]. There was no evidence of an association between deprivation and diagnosis in a UK based study [17], whereas a previous study in England found a positive association between diagnosis rates and deprivation [54]. One study found that there were differences in access to dementia services even in the constituent nations of the UK, with prescribing rates for dementia medication considerably lower in England for those living in deprived areas compared to the rest of the UK [53]. Inconsistencies in findings across studies may relate to geographic location and specific government and healthcare policies, study methodologies, characteristics of the study cohort and the number of people in care homes. In Wales, for example, there has been a relative lack of incentives from and targets set and monitored by the government relating to dementia diagnosis rates focussing on dementia registers maintained by GP practices, compared with England and Scotland, which may contribute to the lower rates of diagnosis [55].

A previous study has looked at the prevalence of neuropsychiatric symptoms in people from England and Wales aged 65 and over, living with and without dementia, where dementia was assessed through the study using the AGECAT algorithm [31]. They found that each symptom was more common in the population living with dementia, except for sleep problems which was common in both groups [31]. We were able to assess whether there were differences in the presence of nine of these symptoms (hallucinations, sleep problems, apathy, delusions, irritability, depression, anxiety, and elation) in people living with undiagnosed dementia, compared to people who had received a diagnosis.

The prevalence of depression in people living with dementia lies around 40% [56] and whilst the association between depression and dementia diagnosis is unclear across other studies [17, 32, 33, 49, 50], we found evidence that those who were experiencing depression may be more at risk of being undiagnosed. There are several possible reasons for this. Low mood may mask dementia symptomatology resulting in a delay in assessment for dementia. It has been shown that depression is most common in the earlier stages of dementia, with the prevalence decreasing as the severity of dementia increases [57–59]. Many people diagnosed with dementia are also prescribed antidepressants to treat the symptoms of depression, which may contribute to the lower prevalence of depression. However, there is some evidence that anti-depressants may not be as effective in people living with dementia as in older people who do not have dementia [60]. In our cohort, 39% of people living with dementia had problems sleeping, which is in line with the 42% reported previously [31]. Despite this increase in prevalence, there was an indication that those with a diagnosis of dementia may be less likely to have sleeping problems compared to those who were undiagnosed. Sleeping problems in people living with dementia are thought to be linked to neurodegeneration and modifications in the brain regions and pathways that initiate and maintain sleep, and regulate sleep–wake cycles [61, 62]. There is an association between sleep and cognitive function [63], and a recent report has found that, over 25 years of follow up, higher risk of late-onset dementia was associated with less sleep in mid-life [64]. The reason for the increased risk of sleep problems in people living with undiagnosed dementia may be because those who have a diagnosis are more likely to receive pharmacological or non-pharmacological interventions specifically targeting sleep, but also because they may have other conditions such as pain and depression which may impact on sleep [65]. In our study sample the majority of people had some symptoms of apathy, in agreement with the study by Savva et al. who found that apathy is more prevalent in those living with dementia compared to those without [31]. There are currently no therapies proven to treat apathy, and apathy severity increases with increasing disease severity and is associated with increased cognitive and functional deficits [66, 67]. We found evidence that more severe apathy was associated with having a diagnosis, likely due to the associated decline in cognition, but these findings suggest that clinicians should also be aware of mild symptoms of apathy when looking for symptoms of dementia. These findings are supported in a US based study, where apathy was associated with increased likelihood of diagnosis [33].

The findings of this study highlight a number of challenges and complexities in diagnosing dementia in the early stages. Firstly, those with early dementia may present with low mood, apathy and insomnia [31], but loss of interest and sleep disorders are also signs of depression [68, 69]. In addition, depression can be related to poor sleep quality [70], which can also affect cognition [71]. Therefore, whilst these symptoms may be indicative of dementia, primary care physicians should be aware that the relationship is complex and be careful not to misdiagnose people. They could use the presence of these symptoms as a tool to help decide whether to do further assessments for dementia.

A second challenge is how to identify and diagnose those who are more highly educated. These people are hypothesised to have a higher cognitive reserve, where the brain can cope with increasing damage whilst still functioning adequately [72]. They are more resilient and can maintain brain function for longer than people with low reserve, and dementia is likely to go undetected for longer, manifesting clinically at a later stage [73, 74]. These theories support other findings that low education is a risk factor for dementia [73, 75, 76]. In England and Wales, screening tests such as the MMSE have been commonly used as part of the assessment for dementia [55]. This has limitations and can result in both an overestimation of the likelihood of dementia in a person with limited education, and an underestimation in those with a higher educational level [55, 77]. Since early signs of dementia are more difficult to detect by standardised tests which only focus on cognition [52], for people who are more articulate or who have a higher IQ, comprehensive neuropsychological assessments by trained psychologists may be needed, but these services are often unavailable.

Inequalities of service provision are another issue, and may affect people in more deprived areas. Factors such as deprivation and urban or rural settings have been associated with variations in local resources and services and health inequality in people living with dementia [78]. A recent report indicates that a third of memory clinics in Wales fail to meet the standard set by the Memory Services National Accreditation program for waiting times [55]. There is no mandatory provision of support in Wales for people with a diagnosis of dementia, which means that access to support is limited for many people, and may ultimately lead to people feeling there is no value in receiving a diagnosis [55]. The diagnosis rates in different areas of the UK vary greatly [4] and in Wales large differences in life expectancy and healthy life persist between the least and most deprived areas [79]. Reduction in social inequality may have a role in increasing diagnosis rates [55].

This study has a number of limitations. The number of people in the CFAS Wales cohort with dementia (either study dementia or a clinical record) was small. People living with dementia may be underrepresented in this cohort because they may be less likely to consent or be less able to complete the interviews and assessments. Many people in the CFAS Wales study did not have an informant taking part, meaning they did not have someone to complete proxy ratings. Since the person living with dementia was the initial contact, for those living with severe dementia accessing an informant willing and able to complete the interviews was difficult. Due to the small sample size, the lack of statistical power in the CFAS Wales cohort was clear for several analyses, with results for many variables having wide confidence intervals and resultant uncertainty in the estimates. Due to the nature of the study, dementia subtype was unknown; it is highly likely that dementia subtype would influence diagnosis, as different subtypes have different characteristics. For example, those with Alzheimer’s Disease or vascular dementia are more likely to experience memory and communication problems, whilst someone with frontotemporal dementia would be more likely to show changes in behaviour which results in greater likelihood of misdiagnosis [80]. There are likely to be very few people with the rarer subtypes of dementia in our cohort. This study also had several strengths. By the nature of undiagnosed dementia, it is not possible to recruit people in this group directly. To overcome this, we undertook comparisons of people living with diagnosed and undiagnosed dementia from a large epidemiological study where participants are not selected based on cognitive impairment, but undergo an assessment of cognitive function suitable for assessment of dementia regardless of whether a clinical diagnosis has been made. We were able to link to official records which included a dementia register, a UK government initiative, to provide information on clinical diagnoses. These studies are challenging and access to data that allows this comparison is rare and a major strength of this study. This study also includes people with all severities of dementia which is difficult given that those with severe dementia may struggle to complete the interviews and assessments. The majority of people in CFAS Wales with study dementia also consented to having their medical records checked, although this could have resulted in exclusion of some of those with more severe impairment who were unable to consent.

The findings of this study suggest that accurate and early diagnosis of dementia is complex, but provides some practical suggestions for clinicians to consider when assessing dementia. In addition to poorer cognitive ability, clinicians should consider education and area-level deprivation, and also the presence of depression, problems sleeping and apathy. However, whilst these symptoms could help clinicians be vigilant in regards to picking up signs of dementia, clinicians should be mindful that they may be related to other illnesses such as depression.

## Conclusions

In summary, our findings suggest that people living with dementia who have better cognition are less likely to be diagnosed, and there is an indication that those who have more years of education, or who live in more deprived areas, are also less likely to have a diagnosis. In terms of neuropsychiatric symptoms, there is some indication that those with depression and sleep problems are more likely to be undiagnosed. Apathy was common across all people living with dementia, but there was some suggestion that severe apathy was more common in those with a diagnosis. The findings of this study may be useful in helping clinicians identify people most at risk of remaining undiagnosed and help them be more aware of signs that could be indicative of early-stage dementia.

## Supplementary Information

**Additional file 1: Table S1**. Description of measures used in this study. **Figure S1**. Selection of those with diagnosed and undiagnosed dementia in CFAS Wales.

## Data Availability

The CFAS Wales datasets analysed during the current study are deposited with the UK Data Service, http://doi.org/10.5255/UKDA-SN-8281-1 [36].

## Abbreviations

AGECAT: Automated Geriatric Examination for Computer Assisted Taxonomy
CFAS Wales: Cognitive Function and Ageing Study Wales
CI: Confidence intervals
GP: General practitioner
MMSE: Mini-Mental State Examination
NHS: National Health Service
OR: Odds ratio
SD: Standard deviation
UK: United Kingdom
US: United States
WIMD: Welsh Index of Multiple Deprivation
