# Supplementary material for: Characteristics of people living with undiagnosed dementia: findings from the CFAS Wales study
Source: BMC Geriatr. 2022 May 10;22:409. doi: 10.1186/s12877-022-03086-4 (PMC9088129; doi:10.1186/s12877-022-03086-4)
Supplement: Supplementary file 1 — Additional file 1: Table S1. Description of measures used in this study. Figure S1. Selection of those with diagnosed and undiagnosed dementia in CFAS Wales. [file 12877_2022_3086_MOESM1_ESM.docx]

**Additional file 1.**

**Supplementary Table 1. Description of measures used in this study**

| Measure | Description |
| --- | --- |
| Sex | Male  Female |
| Age | Continuous (years) |
| Marital status | Married (married/cohabiting)  Not married (single/widowed/divorced/separated) |
| Education | Continuous (years) |
| Living situation | Lives alone  Lives with other (spouse or family/friend)  Lives in care (residential/nursing home) |
| MMSE | 0-17  18-30 |
| Deprivation index | High (top third)  Middle (middle third)  Low (bottom third) |
| Number of health conditions | Angina, intermittent claudication, high/low blood pressure, cancer, diabetes, Parkinson’s disease, stroke, heart attack, fits/epilepsy, serious head injury, chronic bronchitis, asthma, arthritis, peptic ulcers, pernicious anaemia, transient ischemic attack, thyroid problems, meningitis and shingles. A summary score was created by scoring each positive answer as one, except for cancer which was two if in the past and three if a current problem.  Total score ranges from 0-21. |
| Neuropsychiatric symptoms | |
| Hallucinations | Does your imagination play tricks on you?  No  Infrequently (*present*)  Frequently/persistently (*present*/*severe*) |
|  | Observation by interviewer: Hallucinating: behaves as though hears voices or sees visions, or admits to doing so?  Yes (*present*/*severe*)  No |
|  | Has the respondent reported any hallucinations or delusions?  Yes (*present*/*severe*)  No |
|  | Informant: Has s/he been troubled by voices or visions not experienced by others?  Yes (*present*/*severe*)  No |
| Sleep problems | Have you had any trouble sleeping recently?  Yes (*present*)  No |
|  | Informant: does s/he have difficulty getting to sleep?  Yes (*present*)  No |
|  | Informant: is s/he restless or wakeful during the night?  Yes (*present*)  No |
| Apathy | How is your interest in things?  No change  Less infrequent (*present*)  Less persistently (*present*/*severe*) |
|  | Have you had too little energy?  No  Mild listlessness (*present*)  Severe listlessness (*present*/*severe*) |
|  | Have you slowed down in your thinking recently?  No  Mild (*present*)  Severe (*present*/*severe*) |
|  | Informant: Has s/he lost interest in doing things s/he did in the past?  No  Mild (*present*)  Severe (*present*/*severe*) |
| Delusions | Is anyone trying to deliberately harm you?  Yes (*present*/*severe*)  No  Note: if interviewer thought this could be true then this was not counted as a delusion |
|  | Do you sometimes get the feeling that people are laughing at you, or talking about you?  No  Infrequently (*present*)  Frequently/persistently (*present*/*severe*) |
|  | Observation by interviewer: Respondent looks/sounds unduly suspicious;  No  Mild (*present*)  Severe (*present*/*severe*) |
|  | Informant: Is there a tendency to be more suspicious or mistrusting?  No  Mild (*present*)  Severe (*present*/*severe*) |
| Irritability | Have you been more irritable lately?  No  Infrequently (*present*)  Frequently/persistently (*present*/*severe*) |
|  | Informant: Has s/he been more (or less) irritable lately?  No  More irritable (*present*)  Much more irritable (*present*/*severe*)  Less irritable |
|  | Observation by interviewer: hostile or irritable;  Absent  Mild (*present*)  Severe (*present*/*severe*) |
| Depression† | Depressed according to AGECAT algorithm  d0/d1/d2  dn3/dn4/dp3/dp4 (*present*) |
|  | Diagnosis of depression |
| Anxiety ^ | Anxiety according to AGECAT algorithm  an0/an1/an2  an3/an4/an5 (*present*) |
| Elation | Observation by interviewer: uncontrollable short bouts of laughing;  Absent  Mild (*present*) |
|  | Observation by interviewer: infectious gaiety;  Absent  Mild (*present*) |

*Notes:* For neuropsychiatric symptoms, the category that indicates that a symptom is present is labelled (*present*). For hallucinations, apathy, delusions and irritability those with more severe symptoms were also selected, and the category responses that indicate severe symptoms are labelled (*severe*). † For depression, the levels are as follows; d0 - no symptoms, d1-d2 – subclinical depression, dn3-dn4 – neurotic subtype, dp3-dp4 – psychotic subtype. ^ For anxiety, an0 – no symptoms, an1-an2 – subcases of anxiety, an3-an5 – clinical levels of anxiety

**Supplementary Figure 1. Selection of those with diagnosed and undiagnosed dementia in CFAS Wales**

Interviewed

N = 3,593

Excluded from the sample: N=206

NLR: 95; Died: 80; Inactive: 31

Did not consent to medical note review

N=822

N=

Consented to medical note review to check dementia register

N= 2,771

**Undiagnosed dementia**

-study dementia

-not on dementia register

N=105

**Diagnosed dementia**

-study diagnosis

-on dementia register

N=19

Information on dementia register and information on study diagnosis

N= 2,565

Assessed for study dementia: AGECAT: N = 3484

Expert assessment: N=109

Not included in this study:

**No dementia**

-no study dementia

-not on dementia register

N=2392

**Dementia register discrepant cases**

-no study dementia

-on dementia register

N=41

8 people were excluded who appeared to improve over time (study dementia at w1 but not at w2) and MMSE/AGECAT was not indicative of dementia at w2
